# Personalized Pain Medicine: The Clinical Value of Psychophysical Assessment of Pain Modulation Profile

**DOI:** 10.5041/RMMJ.10131

**Published:** 2013-10-29

**Authors:** Yelena Granovsky, David Yarnitsky

**Affiliations:** Department of Neurology, Rambam Medical Center, and Clinical Neurophysiology Lab, Faculty of Medicine, Technion – Israel Institute of Technology, Haifa, Israel

**Keywords:** Conditioned pain modulation, individualized medicine, pain, psychophysics, temporal summation

## Abstract

Experimental pain stimuli can be used to simulate patients’ pain experience. We review recent developments in psychophysical pain testing, focusing on the application of the dynamic tests—conditioned pain modulation (CPM) and temporal summation (TS). Typically, patients with clinical pain of various types express either less efficient CPM or enhanced TS, or both. These tests can be used in prediction of incidence of acquiring pain and of its intensity, as well as in assisting the correct choice of analgesic agents for individual patients. This can help to shorten the commonly occurring long and frustrating process of adjusting analgesic agents to the individual patients. We propose that evaluating pain modulation can serve as a step forward in individualizing pain medicine.

## INTRODUCTION

Since pain is a subjective and complex experience, researchers have found substantial difficulties in measuring it and, consequently, in promoting research into it. One of the common approaches to bypass this difficulty is the use of experimental pain stimuli, given in well-defined and quantitative ways. The measures of pain thresholds and pain tolerance were the main parameters sought over many years. Experience has taught us that thresholds are useful parameters for assessment of sensory deficit, as part of diagnosing nerve damage. This way, elevated thresholds for perceiving the sensations of warm, cold, and mechanical and electrical stimuli are often used in assessing severity of neural damage, such that a high threshold indicates more severe neuropathic damage. This is especially important for damage to small fibers, whose function is not depicted by standard nerve conduction—electromyography tests. Thresholds for painful stimuli, similarly, can identify loss of function, but, since part of the pain inventory of symptoms includes gain of function, such as allodynia and hyperalgesia, these thresholds can be increased or decreased, lowering their sensitivity in identifying the nerve damage. Further, thresholds are not necessarily correlated with the pain experience patients undergo. The best example would be the painful diabetic neuropathy, where the patients demonstrate a combination of peripheral sensory loss and hyperalgesia at the initial stage of disease; in contrast, at the advanced stage the patients exhibit both sensory loss and hypoalgesia, as can be assessed via quantitative sensory testing (QST).

Magnitude estimation of painful stimuli given at supra-threshold intensity is a different approach to the use of experimental stimuli in the pain lab. Practically, a painful stimulus is administered, whose intensity is higher than the pain threshold for that individual, and lower than the pain tolerance. A rating on a visual analog scale (VAS) or a numerical pain score (NPS) is given by the patient. Several studies have shown significant association between supra-threshold pain obtained from patients before surgery, and the levels of their acute post-operative pain.^1^–^6^ More specifically, the association of pre-surgery perception of the experimental pain stimuli and the post-operative pain intensity was established for thermal, mechanical, and electrical sensory modalities in gynecology, back, and knee surgeries, as well as in thoracotomy, cholecystectomy, and herniotomy, including laparoscopy surgeries. However, the above-mentioned parameters of pain threshold, supra-threshold pain estimation, and pain tolerance are usually related to as the static parameters of experimental pain, which isolate a single point of the pain experience of the patient.

**Figure 1:**
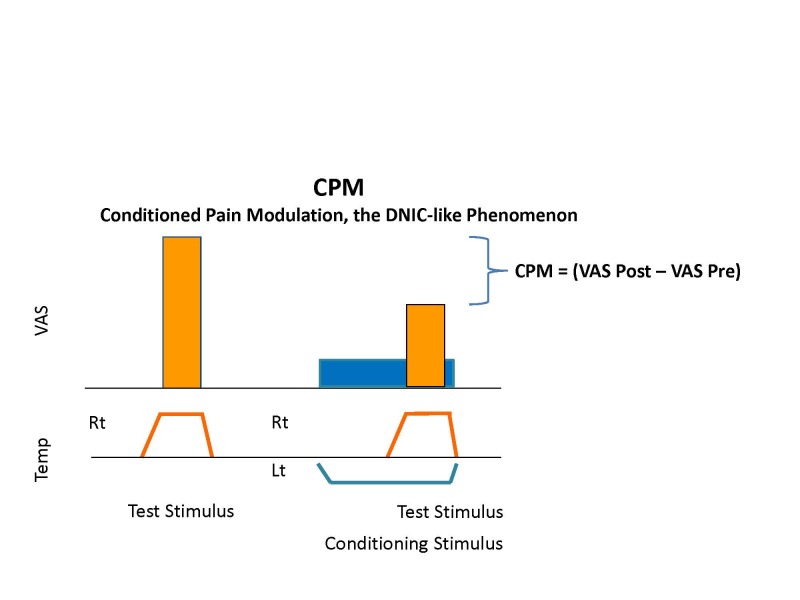
**An Example of a Conditioned Pain Modulation (CPM) Test Protocol.**

A further step forward in pain psychophysics is the use of the dynamic stimulation protocols that give an array of stimuli, in varying combinations, to evoke a process of pain modulation. Pain inhibition is measured by the diffused noxious inhibitory control (DNIC) effect. This is a physiological phenomenon described in the late 1970s in animals, expressing the fact that painful stimuli exert inhibitory effects over other painful stimuli.^7^,^8^ Thus, if we take it to the human pain assessment, if a subject is asked to rate the intensity of a certain test stimulus and then given the combination of a conditioning pain and a repeated same test stimulus, the perceived intensity of the second test stimulus will generally be lower than when given alone. The term conditioned pain modulation (CPM) has recently been coined for the psychophysical protocols^9^ that explore the DNIC phenomenon (Figure 1) and reflects the function of the descending tracts that control and modulate pain perception. These tracts, whose activity is initiated in the brainstem pain-controlling centers, are influenced by cerebral (the top-down effect) as well as up-going painful stimuli (bottom-up) and can exert either inhibition or facilitation on the spinal second-order neurons. Descending pain inhibition that underlies the CPM response is based on a spino-bulbar-spinal loop^7^,^8^,^10^,^11^ that involves serotonergic and noradrenergic neurotransmission.^12^–^14^ The aforementioned neurotransmission construct of the CPM response suggests augmentation of the descending inhibition leading to anti-nociception by increase of synaptic levels of noradrenaline and serotonin.^15^

Pain facilitation is measured using the temporal summation (TS) protocol, where a series of identical stimuli is given and NPS obtained along the series. The common response is an increase in pain ratings along the series, representing the physiological phenomenon of wind-up—the sensitization of nociceptors in response to intense activation. TS represents neurophysiologic processes induced by excessive activation of N-methyl-D-aspartate (NMDA) receptors of the second-order neurons, in response to intensive nociceptive input, and its expression depends on flow of Ca^2+^ ions into the neuronal cytoplasm.^16^ Thus, neuronal wind-up subsequent to the enhanced Ca^2+^ influx-dependent release of glutamate, norepinephrine, and substance P may serve as a target for the agents that are expected to diminish this central neuronal hyperexcitability. In other words, agents that target the Ca^2+^ influx may reduce enhanced TS and alleviate pain.

These dynamic tests induce a process of modulation and are believed to reflect the “real-life” modulation exerted by patients when exposed to clinical pain. There is a large body of data showing differences between pain modulation states in patients suffering from idiopathic and other pain syndromes as compared to the healthy controls:

- *Fibromyalgia*. Various pain modalities applied for the noxious conditioning stimulation, by ischemic, contact heat, or cold noxious water, were non-efficient in increasing pain thresholds or reducing experimental supra-threshold pain magnitudes.^17^–^19^ Evidence for abnormal TS includes enhanced pain summation in response to repeated heat taps and repeated muscle taps delivered at a remote body area, as well as prolonged and enhanced painful after-sensations. Moreover, magnitudes of enhanced after-sensations were predictive of patients’ ongoing clinical pain.^20^–^22^
- *Irritable bowel syndrome*. The experimentally induced visceral or cold water pain was not effective in reducing ongoing rectal pain or the perception of noxious heat.^23^–^25^
- *Headache.* Facilitation, rather than normally occurring inhibition, of nociceptive reflex was observed in migraine patients conditioned by noxious cold water.^26^ In line with this, in chronic tension-type headache patients, conditioning by tonic muscle pain failed to reduce the responses to electrical pain as recorded by somatosensory event-related potentials over the scalp.^27^ In the psychophysical domain, these patients demonstrated less efficient CPM in terms of lower increase in the electrical pain threshold during the exposure to conditioning pain,^28^ as well as significant waning of the CPM at the repeated application.^29^ Increased TS was found in migraine patients for repeated mechanical and electrical noxious stimuli delivered at the periorbital area as well as at a remote body site. Moreover, enhanced TS was demonstrated in association with more severe clinical parameters of disease and tended to normalize with time elapsed since last migraine attack.^30^
- *Temporo-mandibular disorder*. Submaximal effort tourniquet application as the conditioning stimulus was found non-efficient in reducing the clinical pain in these patients.^31^ These patients also responded with increased TS to repeated heat and to repeated mechanical noxious stimuli delivered on local and on remote from the painful body sites.^32^–^35^
- *Osteoarthritis*. Patients with knee and with hip osteoarthritis demonstrated less efficient CPM as assessed by the effect of experimental or ongoing clinical pain on pressure pain thresholds.^36^–^40^ In addition, they demonstrated significant enhancement of TS to noxious pressure as well as to noxious heat stimuli at the site of inflammation and at remote body regions.^41^
- *Whiplash.* Results of a recent study raised evidence for impaired descending pain inhibition in chronic whiplash patients such that the application of ischemic pain as conditioning stimulus did not diminish the perception of pressure pain stimuli.^42^ In line with deficient endogenous pain inhibition, widespread deep tissue hyperalgesia in chronic whiplash was associated with enhanced TS to pressure pain stimuli.^43^,^44^

**Figure 2:**
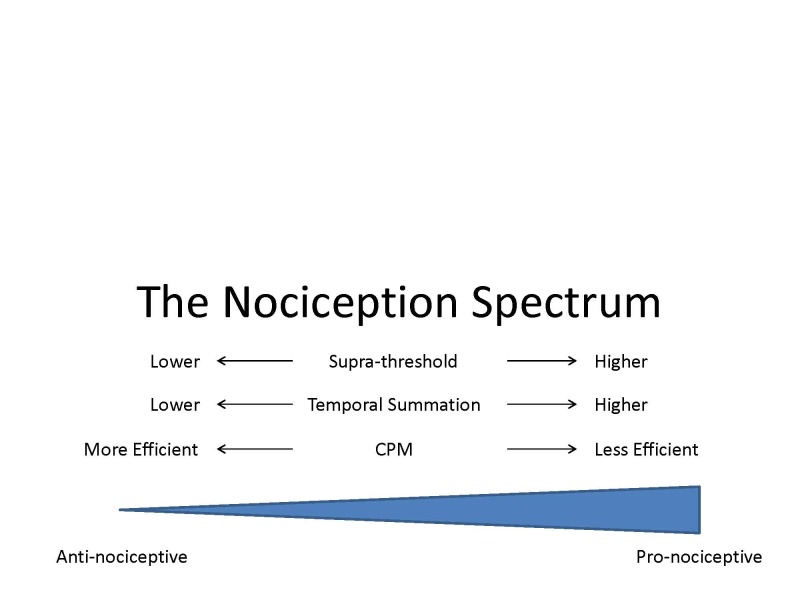
**The Expression of Psychophysical Tests along the Pain Modulation Profile.**

Consequently, the term “pro-nociceptive” is commonly used to describe, at the clinical level, the pain modulation profile of patients suffering from the idiopathic pain disorders. As can be seen from the aforementioned literature overview, these patients can express less efficient CPM, enhanced TS, or both, at psychophysical and neurophysiological levels, as compared to healthy subjects (Figure 2). The exact interrelations between inhibitory and facilitatory pain modulation systems in the clinical arena are still unclear. The reverse situation, an “anti-nociceptive” profile, is less known to us; most likely it represents an inherent or medication-induced resistance to pain. Likely examples would be the pain reduction in migraine patients in response to preventive treatment, and prevention of post-surgical pain by pre-emptive analgesic treatment.

The above-mentioned cross-sectional studies do not disclose whether the interrelations between the modulation state and the presence of the various pain syndromes are causative, and, if so, which one is primary to the other; it could be, on one hand, that a pre-existing facilitatory modulation state leads to the establishment of the pro-nociceptive profile and the acquisition of the idiopathic pain syndromes, or, on the other, that presence of the pain syndrome caused a change in modulation state and profile, shifting it toward the pro-nociception.

In an attempt to discern these potential causative relations, we explored these relationships in a longitudinal study performed in our lab, where pre-thoracotomy, pain-free patients were examined with the battery of psychophysical tests, including assessment of their pain modulation. The patients were followed up 1 year for acquisition of pain after surgery.^45^ The results of this study confirmed our hypothesis that the baseline, pre-surgery CPM efficiency correlated with the intensity of post-operative pain. Moreover, among various demographic and psychophysics parameters (pain thresholds and supra-threshold pain), CPM efficiency was found to be the sole predictor of chronic post-thoracotomy pain such that less efficient CPM patients had higher risk of development of chronic post-surgery pain and higher pain intensity. This reasonably establishes causative relations, at least in one direction, with pain modulation as a pathogenetic factor for future clinical pain. Results were later reproduced by Landau et al. and Wilder-Smith et al. for cesarean section and major abdominal surgery patients, respectively.^46^,^47^ Another interesting piece of evidence supporting “deficient pain inhibition = more pain acquisition” causative relations came from a recent animal-based study that shows the efficient engagement of descending inhibition to be a protection against the development of chronic neuropathic pain.^48^

A further advancement in the lab-to-clinic perusal of pain modulation is in the treatment of pain. Since pain modulation plays a role in pain acquisition, it should affect the relief of pain as well. Our assumption was that pain should be treated by “fixing” the dysfunctional pain modulation parameter of the individual patient. This way, patients with less efficient CPM should benefit more from serotonin-noradrenaline re-uptake inhibitors (SNRIs), which augment descending inhibition by spinal monoamine re-uptake inhibition, than patients whose CPM is already efficient. Similarly, those patients with enhanced TS should benefit more from gabapentinoids, inhibiting central neuronal sensitization, than those with non-enhanced pain summation. We examined CPM and TS in 30 painful diabetic neuropathy patients and found that among other psychophysical factors CPM predicted the efficacy of duloxetine, an SNRI; patients with less efficient pre-treatment CPM expressed high treatment efficacy in terms of pain reduction, while those with efficient CPM did not gain from the drug.^49^ Further, for the former group, an improvement in CPM was found along with pain reduction, while no change in CPM was found for the latter group. Importantly, the CPM remained the only significant predictor for the duloxetine-induced pain relief after controlling for initial clinical pain, pre-treatment level of depression, neuropathy severity, and the placebo effect. On a similar note, Lavand’homme et al. published an abstract in 2009 on the use of ketamine in post-cesarean pain—they found that those patients with enhanced TS to repeated mechanical stimuli, tested pre-operatively, gained more analgesia from ketamine, an NMDA receptor blocker expected to reduce central neuronal sensitization, while those with non-enhanced pain summation did not benefit from the drug.^50^ It thus seems that the dysfunctional modulation state can be instrumental in the choice of drug for pain alleviation. This is a step forward toward individualized pain medicine.

A further question pertaining to pain modulation is whether it is flexible, or unchanged throughout life. A study on osteoarthritis patients undergoing hip replacement surgery showed an improvement in CPM, along with pain alleviation.^36^ It is noted that this was obtained for only one of several CPM protocols used in that study, a finding that highlights the need for additional studies on the interrelations between various testing protocols of pain modulation that yield varying results. Similar results were reported for patients undergoing knee replacement surgery.^39^ These post-surgical results, together with our post-medication results reported above on diabetic neuropathy, suggest that pain modulation is a dynamic feature that probably tends to become pro-nociceptive during pain and to shift back upon alleviation of the pain.

Obviously, a pain modulation profile depends on many factors: 1) genetic factors, 2) environmentally influenced psychosocial factors, 3) the specifications of the pathology generating clinical pain, and 4) the pharmacological agents used to prevent or treat pain. Studies in recent years are trying to integrate psychophysical as well as genetic, neurophysiological, imaging, and other factors in exploring the pain phenomenon. A few recent examples follow: healthy subjects with low expression of serotonin transporter gene demonstrated less efficient CPM effect on pressure pain threshold and noxious heat.^51^,^52^ In the neurophysiology domain, a pain-evoked potentials-based source localization study showed reduced prefrontal cortical activity that was associated with altered pain inhibitory modulation in migraine patients.^53^ A recent neuroimaging study characterized the CPM response as associated with reduced hemodynamic responses in classical pain-responsive areas; furthermore, the CPM efficiency was associated with strength of functional connectivity between various structures on brain endogenous analgesia system.^54^ Finally, there is an important integrative study by Loggia et al. who showed a “triple interaction” between the pain psychophysics, the activation in pain modulatory structures as measured by functional magnetic resonance imaging technique, and the genetics of catecholamine turnover.^55^

Needless to say, further work is required in improving the protocols used for the dynamic psychophysics tests for individual pain assessment, to optimize their reliability, sensitivity, and specificity in describing the clinical pain events, finding the specific test paradigms for specific clinical questions or pathologies, and characterizing the relationships between the various pain testing paradigms. Use of new modalities of exploring the individual pain modulation capabilities, or new neuromodulatory technologies such as repetitive transcranial magnetic stimulation or novel transcranial direct-current stimulation, in combination with psychophysical test paradigms, is a promising new avenue for research in the pain field.

## Abbreviations:

CPM: conditioned pain modulation;
DNIC: diffused noxious inhibitory control;
NMDA: N-methyl-D-aspartate;
NPS: numerical pain score;
QST: quantitative sensory testing;
SNRIs: serotonin-noradrenaline re-uptake inhibitors;
TS: temporal summation;
VAS: visual analog scale.
